# Non-thyroidal illness syndrome and its relationship with mortality risk in critically ill children

**DOI:** 10.3389/fped.2023.1142332

**Published:** 2023-03-03

**Authors:** Laura Carreras, Isolina Riaño, Ana Vivanco, Noelia Avello, Tania Iglesias, Corsino Rey

**Affiliations:** ^1^ Department of Pediatrics, Hospital Universitario Central de Asturias, Oviedo, Spain; ^2^ Department of Pediatrics, University of Oviedo, Oviedo, Spain; ^3^ Health Research Institute of the Principality of Asturias (ISPA), Oviedo, Spain; ^4^ Spain Primary Care Interventions to Prevent Maternal and Child Chronic Diseases of Perinatal and Developmental Origin (RICORS), Institute of Health Carlos III, Madrid, Spain; ^5^ Clinical Biochemistry, Laboratory of Medicine, Hospital Universitario Central de Asturias, Oviedo, Spain; ^6^ Statistical Consulting Unit of the Scientific-Technical Services of the University of Oviedo, Gijón, Spain

**Keywords:** non-thyroidal illness syndrome (NTIS), critically – ill patients, thyroid hormone, inflammatory markers, mortality scores

## Abstract

**Introduction:**

Non-thyroidal illness syndrome (NTIS) is considered to be associated with adverse outcomes in critically ill children.The hypothesis that thyroid hormones and inflammatory markers are associated with increased prediction of mortality risk scores is tested in this paper.

**Methods:**

A prospective observational study was set up in a pediatric intensive care unit (PICU). One hundred and three patients were included. NTIS was defined as a low free triiodothyronine (FT3) value for the patient's age. Thyroid hormones levels and inflammatory markers were determined at admission: FT3, FT4 (free thyroxine), TSH (thyroid-stimulating hormone), rT3 (reverse triiodothyronine), CRP (C-reactive protein) and PCT (Procalcitonin). They were compared between children with a pediatric risk of mortality score PRISM-III >75th percentile (group A, *n*= 25) and the rest (group B, *n* = 78).

**Results:**

A FT4 value lower than 16.6 pmol/L showed an area under the curve (AUC) of 0.655 (0.56–0.78, *p* = 0.02), with 76% sensitivity and 61.5% specificity to detect a high risk of mortality. A multiple regression analysis revealed that a FT4 lower than 16.6 pmol/L [OR: 4.92 (1.60–18.19), *p* = 0.009] and having NTIS [OR: 6.04 (1.45–27.93), *p* = 0.016] could predict a high risk of mortality.

**Conclusions:**

In unselected critically ill children, FT4 and FT3 values at admission could be used as a good predictor of a high mortality risk. We have not achieved a predictive model that combines hormones with inflammatory markers.

## Introduction

Patients in the intensive care unit (ICU) typically present with decreased concentrations of both, plasma free tri-iodothyronine (FT3) and free low thyroxine (FT4), and normal range to slightly decreased concentration of thyroid-stimulating hormone (TSH). This ensemble of changes is collectively known as “Non-thyroidal illness syndrome” (NTIS). Other names for this hormonal disorder are “Low T3 syndrome” or “Sick euthyroid syndrome” (1, 2).

Several clinical studies have shown that the inflammatory cytokines are causally associated with the thyroid hormone metabolism and thus with the NTIS pathogenesis, making it part of the acute phase response (3–5).

It is believed that the pathogenesis of NTIS involves the induction of type III deiodinase (D3), which catabolizes T4 (the prohormone) to rT3 (the inactive hormone) instead of T3 (the active one). Thereby, the increase of D3 activity correlates negatively with serum T3 and with the FT3/rT3 ratio (2). There is also decreased expression of type I deiodinase (D1), which normally converts T4–T3 in the tissues (1, 6, 7). Furthermore, during inflammation tanycytes, specialized cells lining the third ventricle, release type II deiodinase (D2), the main T3 producing enzyme in the brain. The conversion of T4 into T3 in the hypothalamus leads to a decreased production of thyrotropin-releasing hormone (TRH) and the consequent inhibition of the hypothalamic–pituitary–thyroid (HPT) axis (8–10). In addition, there is a decrease in the serum levels of thyroid hormone transport proteins, which inhibits T4 transport in T3-producing tissues (1, 6). All these disorders lead to a drop in plasma T3 levels during acute illness, and especially when there is an inflammatory response.

NTIS seems to be a consequence of the acute phase response to systemic illness and macronutrient restriction, which might be beneficial and should not be treated. However, this is still a controversial topic (1, 6, 9, 11–13).

The extent of NTIS is associated with severity of the disorder and as a result, is associated with prognosis, but no proof exists for causality of this association (2, 14–17), In children, the prognostic value of the syndrome has been reported in the context of sepsis, premature newborns or cardiac postoperative children (18–20), but few studies have focused on the set of diseases which would require Pediatric Intensive Care Unit (PICU) admission (21). Other patterns of NTIS have been described, but in the classic definition decrease in T3 is the main finding, which may or may not be accompanied by a decrease in T4 and/or TSH (1–3).

Regarding inflammatory markers CRP (C-reactive protein) and PCT (Procalcitonin) are the most commonly used in daily clinical practice. CRP rises in response to infectious and inflammatory diseases and shows greater elevations in serious bacterial infections (23–25). It has been shown to be elevated in adult patients with a higher mortality risk (26, 27). Procalcitonin (PCT) was initially used to determine sepsis diagnosis (23) and, afterwards, to help in severity classification of patients (24) and to guide antibiotic treatment duration (28). There are also studies that relate it to a high risk of mortality in children (29).

The objective of this paper is to study if severity of thyroid hormones alteration is independently associated with a higher prediction of mortality risk scores in children. As a secondary objective, the ability of thyroid hormone level together with inflammatory marker concentrations to evaluate increased prediction of mortality risk scores is also tested.

## Material and methods

A prospective observational study was designed without therapeutic intervention. Patients under 18 years old admitted in a University Hospital PICU from June 2018 to February 2020 were included. The exclusion criteria were no blood extraction during the first 24 h, previous thyroid pathology, and parents, guardians or children above 12 years old who did not consent to participate.

The following variables were recorded at admission: age, gender, weight, height, previous ASA scale (American Society of Anesthesiologists) (30), previous chronic treatments, cause of PICU admission (based on American Academy of Pediatrics classification) (31), drugs received during admission, type and time of start of nutrition, evolution (exitus, full or partial recovery) and days of PICU stay. The PRISM III (*Pediatric Risk of Mortality Score*) was calculated during the first 24 h after admission. NTIS was evaluated in every patient, and it was defined following classic criteria, as a FT3 level below the normal value for the age.

Biochemical routine determinations including CRP and PCT were performed at admission. A plasma aliquot was frozen and stored at −80°C for further determination of thyroid hormones (TSH, FT4, FT3 and rT3).

### Mortality risk groups

Patients were divided into two groups according to a mortality risk score. The PRISM III scale was chosen as it is the most commonly used in our daily clinical practice (32–34). The higher risk mortality score group (Group A) included patients with PRISM III >p75 (*n* = 25); the lower risk mortality score group (Group B) included patients with PRISM III ≤p75 (*n* = 78). This risk-based classification has already been used in other studies, given that mortality in pediatrics is generally not high (29).

### Measurement of TSH, FT4, FT3, rT3

TSH, FT4 and FT3 were measured in plasma by electrochemiluminescence immunoassay (ECLIA) on Cobas analyzer e801 (Roche Diagnostics GmbH, Mannheim, Germany). The reference values were those used by the laboratory where they were performed, and these values were age-dependent (35). Plasma rT3 levels were measured with RIA (DIAsource ImmunoAssay, Belgium), and its normal values were based also on patient's age (36).

PCT was measured in lithium-heparin plasma by ECLIA on Cobas analyzer e601 (Roche Diagnostics GmbH, Mannheim, Germany). Analytical detection limit was 0.02 ng/ml. Plasma CRP was measured on a Modular Analytics Cobas 6,000 (Roche diagnostics) by an immunoturbidimetric technique, and the analytical detection limit was 0.07 mg/dl.

### Statistical analysis

A descriptive analysis was performed. Qualitative variables were described using relative and absolute frequencies, and quantitative variables using position and dispersion measurements. Differences between the two groups were assessed with Student's *t*-test (with Welch's correction for different variances) or Wilcoxon's test for independent samples, depending on whether or not the normality hypothesis was met. Pearson or Spearman correlations were calculated, depending on whether or not normality was verified, for quantitative variables.

The FT4 and FT3 levels, the FT3/rT3 index and the biomarkers (CRP and PCT) were evaluated for predicting high mortality risk on the PRISM III score. Optimal cut-off points were calculated according to the Youden index, which simultaneously maximizes Sensitivity (Se) and Specificity (Sp). Cut-off points, Sensitivity, Specificity, positive predictive value (PPV), negative predictive value (NPV), area under the ROC curve (AUC) and the significance of the tests were also provided.

Taking into account the optimal cut-off points previously calculated, univariate and multivariate binary logistic regression models were constructed to predict high risk values on the mortality scale. Odds ratio (OR) were provided with their 95% confidence intervals. Goodness-of-fit was assessed through the likelihood-ratio test, AUC and Nagelkerke's coefficient *R*^2^. The statistical significance level used was 0.05. R Program (R Development Core Team) version 3.6.3 was used for the statistical analysis (37).

### Statement of ethics

Written informed consent was obtained from patients’ parents or guardians and from children above 12 years old. The blood for analyzing hormone levels was obtained and frozen from the blood sample used in routine laboratory tests; therefore, it was not necessary to obtain an extra sample. The information collected from each patient was treated in a blinded manner, using a code to identify each one.

The study protocol was approved by the Clinical Research Ethics Committee of the Autonomous Region of Asturias. All procedures performed in the study were in accordance with the ethical standards of the institutional ethics committee and with the 1964 Helsinki declaration and its later amendments.

## Results

### Baseline characteristics

A total of 251 consecutive patients were enrolled over a 21-month period. Ninety-three were not elegible due to lack of informed consent (not requested by physicians or not signed by parents or guardians); 2 were excluded because of thyroid pathology (not previously known); 4 because they did not have blood tests performed in the PICU (only in the Emergency room); and 8 for insufficient blood sample for the analysis. Of the remaining 145 patients, only those with blood tests within the first 24 h of admission were selected for this study. The final sample was 103 children (54% male, mean age 6.64 ± 5.20 years).

Baseline demographic and clinical data of the two groups (higher and lower score risk mortality) are shown in Table 1. There was no difference in the previous health state (ASA scale and need of chronic treatments), in the PICU stay nor in the further evolution. Hemato-oncological and multisystemic disease (mainly sepsis and polytrauma) were significantly the most frequent cause of admission in the high risk group. There was no difference in the timing of nutrition initiation between the two groups. Patients in group A received more often inotropic drugs, furosemide and blood products. The NTIS prevalence in the overall population was 30.1%, but no differences were found between groups.

**Table 1:** Demographic and clinical data.

	Group A (*n* = 25)	Group B (*n* = 78)	*p*-value	Overall population (*n* = 103)
**Sex**				
Female	10 (40.0)	37 (47.44)	0.675	47 (45.63)
Male	15 (60.0)	41 (52.58)		56 (54.36)
**Age (years)**	8.51(2.19–11.62)	5.78 (1.13–11.39)	0.27	6.64 (1.35–11.54)
**Somatometry**				
Weight (kg)	29.10 (16.99)	25.60 (18.66)	0.406	26.30 (18.22)
Height (cm)	119.64 (31.85)	109.47 (37.57)	0.254	111.36 (36.55)
**Previous ASA score**				
ASA 1	17 (68.0)	52 (66.67)	0.827	69 (66.99)
ASA 2	5 (20.0)	11 (14.10)		16 (15.53)
ASA 3	3 (12.0)	12 (15.38)		15 (14.56)
ASA 4	0 (0.0)	3 (3.85)		3 (2.91)
**Chronic treatments**				
No	20 (80.0)	62 (79.49)	1	82 (79.61)
Yes	5 (20.0)	16 (20.51)		21 (20.38)
**Admission diagnosis**				
Postoperative	2 (8.0)	29 (37.18)	0.02	32 (31.07)
Respiratory	7 (28.0)	22 (28.21)		29 (28.15)
Multisystemic	7 (28.0)	10 (12.82)		17 (16.50)
Nervous system	3 (12.0)	8 (10.26)		11 (10.68)
Hemato-oncological	3 (12.0)	3 (3.85)		6 (5.83)
Others	2 (8.0)	6 (7.68)		8 (7.77)
**Nutrition initiation**				
Early nutrition	19 (76.0)	61 (78.21)	1	80 (77.66)
Late nutrition	6 (24.0)	17 (21.79)		23 (22.33)
**Treatments in PICU**				
Benzodiazepines	11 (44.0)	23 (29.49)	0.272	34 (33.0)
Corticosteroids	8 (32.0)	16 (20.51)	0.363	24 (23.30)
Opioids	9 (36.0)	21 (26.92)	0.538	30 (29.13)
Inotropics	8 (32.0)	0 (0.0)	<0.001	8 (7.76)
Furosemide	7 (28.0)	4 (5.13)	0.004	11 (10.68)
Blood products	11 (44.0)	9 (11.54)	0.001	20 (19.42)
**Evolution**				
Exitus	2 (8.0)	7 (8.97)	0.531	9 (8.70)
Recovery	23 (92)	71 (91)		94 (91.3)
**PICU stay (days)**	4.0 (2.0–7.0)	3.00 (2.00–5.75)	0.208	4.59 (2.0–6.0)
**NTIS (first 24 h)**	11 (44.0)	20 (25.65)	0.136	31 (30.10)

Group A: Higher score risk mortality group. Group B: Lower score risk mortality group.

Weight and height are described by average (standard deviation); Age and PICU stay are described by median (P25–P75); the rest of variables are described by the absolute and relative values (%).

Nutrition initiation refers to both enteral and parenteral; Early: started in the first 24 h of admission; Late: started later.

### Mortality scores

Mean value and standard deviation of PRISM-III score at admission was 3.94 (6.27). The 75th percentile of PRISM-III (6 points) was the one used to divide the total sample in group A and B.

### Hormone levels and inflammatory markers

As can be seen in Table 2, there were no differences in CRP and PCT levels between both groups. The decline of TSH, FT3 and FT4 was greater in the high risk group, although FT3 did not reach statistical significance. There was no difference in the rT3 elevation or in the FT3/rT3 index.

**Table 2:** Laboratory data.

	Group A	Group B	*p*-value	Overall population
**Inflammatory markers**				
CRP	0.80 (0.20–2.30)	1.70 (0.40–6.00)	0.216	5.88 (13.01)
PCT	0.47 (0.11–2.26)	0.22 (0.08–0.92)	0.216	2.34 (5.79)
**Hormones levels**				
TSH	1.37 (0.85)	2.35 (1.78)	<0.001	2.11 (1.65)
FT4	15.31 (3.99)	17.37 (3.47)	0.018	16.86 (3.73)
FT3	3.99 (1.72)	4.65 (1.61)	0.082	4.50 (1.64)
rT3	502 (425–901)	772 (476–978)	0.103	759 (438)
FT3/rT3 index	4.75 (3.07–12.86)	4.63 (3.09–8.44)	0.768	7.63 (7.79)

Group A, Higher score risk mortality group; Group B, Lower score risk mortality group.

CRP, c-reactive protein (mg/dl); PCT, procalcitonin (ng/ml); TSH, thyroid-stimulating hormone (mIU/L); FT4, free thyroxine (pmol/L); FT3, free triiodothyronine (pmol/L); rT3, reverse triiodothyronine (pmol/L); FT3/rT3 index was obtained according to the traditional units (pg/ml for FT3 and ng/ml for rT3).

Variables that do not meet the hypothesis of normality (CRP, PCT, rT3 and FT3/rT3 index) are expressed as median (P25–P75); the other variables are described by mean (SD).

In the overall population column, variables are expressed as median (SD).

### Logistic regression models

A binary logistic regression model was constructed to predict high risk prediction on the PRISM III score, as a function of CRP, PCT, FT3, FT4 and FT3/rT3 index. Previously, the optimal cut-off point was calculated for each one, as shown in Table 3. FT4 cut-off (<16.6 pmol/L) was the only one that significantly discriminated higher risk, with an AUC of 0.655 (0.53–0.78). The other variables were included in the model without using a cut-off point. NTIS was also added to the model as a predictor variable.

**Table 3:** Optimal cut-off points and accuracy indicators for inflammatory biomarkers and hormones to differentiate higher and lower score risk mortality groups.

	Cut-off point	Se (%)	Sp (%)	PPV (%)	NPV (%)	AUC (CI 95%)	*p*-value
CRP	<2.3	76.0	44.1	30.7	30.7	0.583 (0.45–0.715)	0.216
PCT	>0.37	60.0	59.7	30.8	83.3	0.592 (0.443–0.741)	0.216
FT4	<16.60	76.0	61.5	38.8	88.9	0.655 (0.53–0.78)	0.02
FT3	<3.44	48.0	79.5	42.9	82.7	0.599 (0.46–0.739)	0.137
FT3/rT3	>11.61	32.0	81.6	36.4	78.5	0.520 (0.379–0.661)	0.768

CRP, c-reactive protein (mg/dl); PCT, procalcitonin (ng/ml); FT4, free thyroxine (pmol/L); FT3, free triiodothyronine (pmol/L); rT3, reverse triiodothyronine (pmol/L); FT3/rT3 index was obtained according to the traditional units (pg/ml for FT3 and ng/ml for rT3).

Se, sensitivity; Sp, specificity; PPV, positive predictive value; NPV, negative predictive value; AUC, area value under the ROC curve.

Table 4 shows the odds ratios, their 95% confidence intervals and the corresponding *p*-value for each univariate regression model. The last column is the multivariate model resulting from including the previous variables in a single model and simplifying it through a step-by-step selection algorithm. FT4 value lower than 16.6 pmol/L increases the mortality risk 5.15 times. Having NTIS (which was defined by a low FT3 value for the patient's age) increases the mortality risk 6.04 times. A high PCT value was also statistically significant, but the OR (1.16) lacks clinical relevance. CRP, FT3 and FT3/rT3 index were not statistically significant neither using univariate nor multivariate analysis.

**Table 4:** Odds ratio (OR) of the univariate and multivariate regression models.

	Group A	Group B	Univariate OR	Multivariate OR
CRP	4.4 (7.8)	6.4 (14.4)	0.98 (0.92–1.02, *p* = 0.508)	0.91 (0.81–0.99, *p* = 0.061)
PCT	5.2 (10.3)	1.5 (3.3)	1.10 (1.01–1.21, *p* = 0.037)	1.16 (1.04–1.34, *p* = 0.017)
FT3	2.6 (1.1)	3.0 (1.0)	0.67 (0.41–1.04, *p* = 0.086)	–
FT4 > 16.6 pmol/L	7 (12.5)	49 (87.5)	–	–
FT4 < 16.6 pmol/L	18 (38.3)	29 (61.7)	4.34 (1.68–12.35, *p* = 0.004)	5.15 (1.51–21.76, *p* = 0.014)
FT3/rT3	8.7 (9.6)	7.3 (7.2)	1.02 (0.96–1.08, *p* = 0.463)	1.06 (0.98–1.15, *p* = 0.143)
No NTIS	14 (19.4)	58 (80.6)	–	–
Yes NTIS	11 (35.5)	20 (64.5)	2.28 (0.88–5.85, *p* = 0.086)	6.04 (1.45–27.93, *p* = 0.016)

CRP, c-reactive protein (mg/dl); PCT, procalcitonin (ng/ml); FT3, free triiodothyronine (pmol/L); FT4, free thyroxine (pmol/L); FT3/rT3 index was obtained according to the traditional units (pg/ml for FT3 and ng/ml for rT3). NTIS, Non-thyroidal illness syndrome.

FT4 cut-off (<16.6 pmol/L) was the only one that significantly discriminated higher risk. The other variables were included in the model without using a cut-off point (only with mean and standard deviation).

This model satisfies the requirement of goodness of fit, with a significant likelihood ratio test (*p* < 0.001), an AUC = 0.826 and a Nagelkerke's coefficient *R*^2 ^= 34.7%.

## Discussion

An important issue in pediatric critical care is to improve prognostic assessment in the first hours after admission. The better evaluated tools have been scales developed to estimate the mortality risk based on clinical and analytical findings. One of the most used scales in critically ill children is Pediatric Risk of Mortality III (PRISM III) (34). Lately, biochemical tests that can be determined in a short time after admission are being studied (29).

To date, few studies have linked NTIS with mortality in unselected critically ill children (21, 22), and to our knowledge, this prospective study is one of the first trying to assess thyroid hormone levels as predictors of outcome in daily clinical practice. We have seen that low levels of FT3 and FT4 are associated with increased risk of mortality scores in a heterogeneous sample of critically ill children.

We couldn't use mortality as the gold standard to differentiate the patients' prognosis because the mortality was low in our sample. Therefore, we have used the PRISM III score as one of the standard PICU outcome tools.

### Comparative analysis

Children with higher or lower mortality risk in the PRISM III score (group A and B) barely show significant differences between them. There were only differences in the admission diagnosis and in the drugs used during their PICU stay. The higher risk group had more children with multisystemic and hemato-oncologic disease, but fewer postoperative patients. This seems logical as it has been described that children with sepsis, polytrauma and with complications derived from an oncologic disease have a worse prognosis (38–40).

In addition, in the higher mortality group, inotropic drugs, furosemide and transfusions were more frequently required. It should be noted that these drugs can interfere in the metabolism of thyroid hormones (1, 6) and, therefore, could influence the NTIS development. However, blood samples were obtained from patients during the first 24 h after admission. Before they were obtained, most of the patients had been given these drugs for a very short period of time.

We found no differences between the groups in terms of nutrition initiation (before or after 24 h of admission). This is interesting because fasting could act as a confounding factor, since it favors the development of NTIS (5, 8).

There were no significant differences in mortality or length of PICU stay. Considering that mortality in pediatrics is not so high, a larger sample size would be necessary to find results that better reflect the real situation. On the other hand, it is not uncommon for certain more severe patients to improve in a short period of time, reducing the time of hospitalization.

In terms of hormone levels, higher-risk patients were noted to have lower plasma levels of FT3, FT4 and TSH, although the FT3 hardly reached statistical significance. These results are consistent with other pediatric studies (18, 19, 21, 22). It should also be noted that the prevalence of NTIS in the total sample is clinically relevant (30%). It seems that the trend is towards a higher prevalence in group A (44%) than in group B (26%), although it does not reach statistical significance (Table 1).

Against expectations, neither a greater elevation of rT3 nor a decrease in the FT3/rT3 ratio was found in the higher-risk group. We believe that early sample collection (first 24 h of hospitalization) may influence this result. It would be useful to see the levels evolution in the following days of admission (1, 2).

Concerning the inflammatory markers, we found no significant differences in CRP concentrations between group A and B. Slow CRP kinetics could explain this finding because our analysis was performed so early. PCT also did not differ between the two groups. Taking into account that it is a biomarker used to detect earlysepsis, this heterogeneous sample of patients is probably not the most adequate to identify a significant increase in PCT in the most severe patients. It would be very interesting to design a specific study of patients with sepsis, in which the increase in PCT could probably be related not only to severity but also to the development of NTIS (23–25).

### High-risk score mortality prediction

Based on the logistic regression model, we have found that children with a FT4 below 16.6 pmol/L and with NTIS at admission (meaning with a low FT3 for their age) are more likely to be at high risk of mortality. We consider that these determinations are available in daily clinical practice and could provide additional prognostic information.

The PCT odds ratio is also a predictor of high risk in the multivariate model, although with a value that lacks clinical significance (OR 1.16). However, it's interesting to see that the results are in agreement with those observed in other pediatric studies (29).

The explanatory power of the multivariate model is 34.7%, which means that other factors may influence the high risk, and could be taken into account in the future as predictors.

### Limitations

Our study presents some limitations. First, we have conducted an observational study that does not allow us to draw conclusions leading to therapeutic interventions. Second, as it was not sufficiently powered to detect differences in survival, we had to use a surrogate marker of mortality (PRISM III score). This means that the cut-off point for high and low risk is determined by our own sample. Third, some of the used drugs (furosemide, inotropics, transfusions) and underlying deseases (as hemato-oncological) could influence the NTIS development. Fourth, it is a single-centre study and, although we have tried to include all types of diagnoses on admission, we have no postoperative cardiac surgery patients in our center. Fifth, the analysis of thyroid hormones included in this paper took place only in the first 24 h of admission. This should be taken into account since the alteration in hormone levels and the prevalence of NTIS varies depending on the time of acute illness (5, 6). In subsequent studies, it would be very useful to measure hormone levels at different times in the same patient. On the other hand, an early severity prediction is more useful in clinical practice in improving patient outcomes. Finally, it should be noted that a considerable number of patients were lost due to a lack of informed consent (unsigned or unsolicited).

Future studies are needed to explore if the association of non-thyroidal illness syndrome with a pediatric mortality risk score represents a maladaptive response that needs to be treated.

## Conclusions

NTIS was associated with an increased prediction of mortality risk score. FT4 lower than 16.6 pmol/L at admission, combined with a low FT3 level for the age of the patient, could be used by clinicians to identify critically ill children at a higher prediction of a death risk score. We have not achieved a predictive model that combines hormones with inflammatory markers.

## Data Availability

The original contributions presented in the study are included in the article/Supplementary Material, further inquiries can be directed to the corresponding author.
